# Cementless total hip arthroplasty for primary osteoarthritis in patients aged 55 years and older: Results of the 8 most common cementless designs compared to cemented reference implants in the Finnish Arthroplasty Register

**DOI:** 10.3109/17453671003635900

**Published:** 2010-03-31

**Authors:** Keijo T Mäkelä, Antti Eskelinen, Pekka Paavolainen, Pekka Pulkkinen, Ville Remes

**Affiliations:** ^1^Department of Orthopaedics and Traumatology, Turku University Central Hospital, TurkuFinland; ^2^Coxa Hospital for Joint Replacement, TampereFinland; ^3^Orton Orthopaedic Hospital, Invalid Foundation, HelsinkiFinland; ^4^Department of Public Health, University of HelsinkiFinland; ^5^Department of Orthopaedics and Traumatology, Helsinki University Central Hospital, HelsinkiFinland

## Abstract

**Background:**

Cemented total hip arthroplasty has been the treatment of choice for elderly patients with osteoarthritis. We analyzed survival rates of the most common cementless designs used in this age group in Finland.

**Patients and methods:**

Inclusion criteria permitted 10,310 replacements (8 designs) performed in patients aged 55 years or older to be selected for evaluation. The risk of revision of each of the 8 implants was compared with that of a group comprising 3 cemented designs as the reference (9,549 replacements). Survival analyses were performed overall and separately for 3 age cohorts: 55–64 years (6,781 replacements), 65–74 years (8,821 replacements), and 75 years or older (4,257 replacements).

**Results:**

In all patients aged 55 years or more, the Bi-Metric stem had a higher survival rate for aseptic loosening at 15 years than the cemented reference group: 96% (95% CI: 94–98) vs. 91% (CI: 90–92). However, the 15-year survival rates of the Bi-Metric/Press-Fit Universal (71% (CI: 67–75)) and the Anatomic Mesh/Harris-Galante II (72% (CI: 67–78)) total hip replacements were lower than that of the reference group (86% (CI: 84–87)). Information was scarce for patients aged 75 years or more.

**Interpretation:**

Cementless proximal porous-coated stems are a good option for elderly patients. Even though biological fixation is a reliable fixation method in THA, polyethylene wear and osteolysis remain a serious problem for cementless cup designs with unplugged screw holes and low-quality liners.

## Introduction

Results obtained from the Scandinavian arthroplasty registries (Havelin et al. 2000, Puolakka et al. 2001a, Malchau et al. 2002) on a nation-by-nation basis and studies from single centers worldwide (Berry et al. 2002, Wroblewski et al. 2002, Della Valle et al. 2004, Buckwalter et al. 2006, Morshed et al. 2007) have indicated that cemented total hip replacement is the treatment of choice for severe osteoarthritis in elderly patients. However, in a recent study based on data obtained from the Finnish Arthroplasty Register, cementless implants were found to have similar long-term survival rates as cemented implants in patients aged 55 years or more (Mäkelä et al. 2008a). Several studies (Archibeck et al. 2001, Bojescul et al. 2003, Jacobsen et al. 2003, Marshall et al. 2004, Meding et al. 2004, Oosterbos et al. 2004, Parvizi et al. 2004) have shown that the survival rates of cementless stems have been satisfactory for all age groups, but cementless cups have a common problem of liner wear, osteolysis, and high incidence of revision in the medium-to-long term (Barrack et al. 1997, Malchau et al. 1997, Havelin et al. 2002, Duffy et al. 2004).

We therefore separately analyzed the survival rates of the most common cementless designs performed for primary osteoarthritis in patients aged 55 years or older in Finland, and compared the risk of revision for each implant with that of the cemented implant reference group (Mäkelä et al. 2008b). These analyses were carried out on population-based data obtained from the Finnish Arthroplasty Register for the period 1980 through 2005.

## Patients and methods

Since 1980, data on total hip replacements have been collected and archived in the Finnish Arthroplasty Register (Paavolainen et al. 1991, Puolakka et al. 2001a). Healthcare authorities, institutions, and orthopedic units in Finland are obliged to provide the National Agency for Medicines with information that is essential for monitoring past and current trends for the efficacious use of materials, approaches, and designs used in orthopedics. The coverage in the Finnish Arthroplasty Register was initially analyzed for the period 1994–1995 by comparing its data with those of the discharge registers of participating hospitals; the Register covered 90% of all implantations performed (Puolakka et al. 2001a). Since 1995, the data in the register have been compared with those of hospital discharge registers every few years. Currently, 98% of implantations are recorded in the Finnish Arthroplasty Register (Peltola 2008).

### Study population and inclusion criteria

During the study period (1980–2005), 101,720 primary total hip replacements were performed in Finland. Of these, 87,578 (86%) were performed on patients aged 55 years or older. Primary osteoarthritis was the indication in 71,146 (81%) of these operations; cementless total hip implants were used in 30,112 (42%).

Only designs used in more than 500 operations during the study period and designs with more than 20 hips at risk at 5 years were included in the current study. These criteria permitted the inclusion of 8 designs (10,310 replacements) (Table 1). The risk of revision for each design was compared with that for 9,549 cemented reference implants (Table 2). A 10-year survival rate exceeding 90% is commonly regarded as a good long-term outcome (National Institute of Clinical Excellance, NICE). The 3 best performing cemented designs in Finland (Mäkelä et al. 2008b) fulfilled this criterion and were chosen as reference implants. These 3 cemented designs were the Exeter Universal stem combined with the All-poly cup (Stryker, Mahwah, NJ), the Müller Straight stem combined with the Müller Standard cup (Zimmer, Warsaw, IN), and the Lubinus SP II stem combined with the Lubinus IP cup (Waldemer Link, Hamburg, Germany). Survival analyses were performed for the whole study population and separately for each of 3 age cohorts: 55–64 years, 65–74 years, and 75 years and older. The data from subgroup analysis were massive and only the data with “any reason” as cause of revision are presented (Table 7).

**Table 1.** Demographic data of the implants analyzed

THR Brands	No.	Mean follow-up	Mean age	Women	(%) No. of hospitals	Period of implantation
Anatomic Mesh/HG-II	604	11.1	63	56	24	1989–1997
PCA Std/PCA Pegged	508	11.6	63	55	23	1985–1995
Bi-Metric/PFU	2,687	8.8	63	49	53	1986–2001
Bi-Metric/Mallory	637	8.7	67	60	11	1989–2000
Bi-Metric/Vision	2,055	3.4	65	48	47	1998–2005
ABG I/ABG I	565	9.1	65	55	25	1992–1997
ABG I/ABG II	1,765	5.9	66	51	36	1996–2003
ABG II/ABG II	1,489	2.5	67	55	31	2000–2005
Cemented reference	9,549	8.8	72	66	62	1980–2005
Together	19,859	7.6	68	59	77	1980–2005
HG-II: Harris-Galante II; PCA Std: porous-coated Anatomic Standard; PFU: Press-Fit Universal; ABG: Anatomique Benoist Girard.						

**Table 2.** Material, surface, design features, and manufacturer of the implants. For abbreviations, see Table 1

THR Brands	Material	Surface	Special design features	Manufacturer
*Stems*				
Bi-Metric	Titanium alloy	Proximally porous-coated	Straight, collarless	Biomet
Anatomic Mesh	Titanium alloy	Proximally porous-coated	Anatomic	Zimmer
ABG I	Titanium alloy	Proximally grit-blasted and HA-coated	Anatomic	Stryker Howmedica
ABG II	Titanium alloy	Proximally grit-blasted and HA-coated	Anatomic	Stryker Howmedica
PCA Standard	CoCr alloy	Proximally porous-coated	Anatomic	Stryker Howmedica
Exeter Universal	Stainless steel	Polished	Straight, collarless, cemented	Stryker Howmedica
Müller Straight	CoCr alloy	Matt	Straight, small collar, fluted macrostructure	Zimmer
Lubinus SP II	CoCr alloy	Matt	Anatomic, collar, modular	Link
*Cups*				
ABG I	Titanium alloy	Grit-blasted and HA-coated	Hemispherical, open screw-holes	Stryker Howmedica
ABG II	Titanium alloy	Grit-blasted and HA-coated	Hemispherical, screw-holes plugged	Stryker Howmedica
Biomet Mallory	Titanium alloy	Porous-coated	Hemispherical, open screw-holes, fins	Biomet
Biomet Universal	Titanium alloy	Porous-coated	Hemispherical, open screw-holes	Biomet
Biomet Vision	Titanium alloy	Porous-coated	Hemispherical, screw-holes plugged	Biomet
Harris-Galante II	Titanium alloy	Porous-coated	Hemispherical, open screw-holes	Zimmer
PCA Pegged	Cobalt-chromium	Porous-coated	Hemispherical, open screw-holes	Stryker Howmedica
Exeter All-poly	Polyethylene	–	Cemented	Stryker Howmedica
Müller Std	Polyethylene	–	Cemented	Zimmer
Lubinus IP	Polyethylene	–	Groove design	Link

Revisions were linked to the primary operation by using the patient's personal identification number; these numbers are assigned to every resident of Finland. Numbers and indications for revision were recorded (Table 3).

**Table 3.** Reasons for revision of the 8 most common cementless brands and the cemented reference designs. Percentage in parentheses. For prosthesis types, see Table 1

A	B	C	D	E	F	G	H	I	J	K	L
Anatomic Mesh/HG-II	604	17 (15)	23 (20)	17 (15)	0 (0)	4 (4)	7 (6)	4 (4)	2 (2)	39 (35)	113
PCA Std/PCA Pegged	508	19 (14)	81 (60)	19 (14)	2 (2)	1 (1)	2 (2)	0 (0)	1 (1)	10 (7)	135
Bi-Metric/PFU	2,687	26 (8)	67 (19)	10 (3)	9 (3)	57 (17)	14 (4)	16 (5)	15 (4)	131 (38)	345
Bi-Metric/Mallory	637	5 (8)	11 (17)	3 (5)	1 (2)	12 (19)	3 (5)	5 (8)	3 (5)	21 (33)	64
Bi-Metric/Vision	2,055	11 (16)	1 (1)	6 (9)	8 (12)	28 (41)	5 (7)	0 (0)	4 (6)	6 (9)	69
ABG I/ABG I	565	10 (9)	27 (26)	3 (3)	1 (1)	6 (6)	3 (3)	3 (3)	6 (6)	47 (44)	106
ABG I/ABG II	1,765	1 (2)	6 (10)	2 (3)	5 (8)	13 (21)	10 (16)	1 (2)	10 (16)	15 (24)	63
ABG II/ABG II	1,489	2 (4)	1 (2)	3 (6)	3 (6)	10 (20)	7 (14)	4 (8)	19 (37)	2 (4)	51
Cemented reference	9,549	227 (28)	142 (18)	253 (32)	38 (5)	73 (9)	20 (3)	5 (1)	24 (3)	16 (2)	798
Together	19,859	318 (18)	359 (21)	316 (18)	67 (4)	204 (12)	71 (4)	38 (2)	84 (5)	287 (17)	1,744
A THR brand											
B No. of primary operations											
C Aseptic loosening (cup + stem)											
D Aseptic loosening (cup)											
E Aseptic loosening (stem)											
F Infection											
G Dislocation											
H Malposition											
I Fracture of the prosthesis											
J Periprosthetic fracture											
K Any other reason											
L Together											

### Statistics

The endpoint for survival was defined as revision when any component (including femoral head and liner) or the whole implant was removed or exchanged. Survival rates for stems and cups were analyzed separately with revision for aseptic loosening being used as the endpoint. When survival analyses were conducted for total hip replacements (cup + stem combinations), both revision for any reason and revision for aseptic loosening served as discrete endpoints. Kaplan-Meier survival analysis was used to calculate the survival probabilities of implants at 7, 10, and 15 years. The survival rate of any respective implant was determined only when there were at least 20 hips at risk at the follow-up point (Furnes et al. 2001). Patients who had died or emigrated from Finland during the follow-up period were censored at that point. Survival data obtained by Kaplan-Meier analysis were compared by the log-rank test. The Cox multiple regression model was used to study differences between implants and to adjust for potential confounding factors.

Both Kaplan-Meier and Cox regression are methods based on assumptions of independent observations. However, bilateral observations cannot be regarded as being independent (Robertsson and Ranstam 2003, Bryant et al. 2006). Violation of this independence assumption may have an effect on the validity of the results. To avoid this violation, the data analysis could be performed by allowing inclusion of correlated observations, e.g. including only one prosthesis per patient or by including a shared frailty variable in the Cox regression. In the current study, however, bilateral observations were included in the dataset analyzed. It has been found that the effect of neglecting bilateral prostheses is minute (Havelin et al. 1995, Robertsson and Ranstam 2003, Lie et al. 2004).

Risk of revision ratios of stems, cups, and total hip replacements (cup + stem combinations) were analyzed. Adjustments were made for age and sex. The 3 best performing cemented designs in Finland were chosen as reference implants (Mäkelä et al. 2008b). The survival data of these cemented designs were combined to form a single reference group. Cox regression analyses provided survival probabilities and adjusted risk ratios for revision. Estimates derived from the Cox analyses were used to construct adjusted survival curves at mean values of the risk factors. The proportional hazards assumption of the Cox model (meaning that the relative difference between revision rates should be constant over time since the primary operation) was not reached in some analyses performed. Thus, adjusted risk ratios were also established within time intervals (0–7 years, 7 years after the primary operation). The Wald test was used to calculate the p-values for data obtained from the Cox multiple regression analyses. A difference between groups was considered to be statistically significant if the p-value was less than 0.05 in a two-tailed test.

## Results

### Survival of stems – aseptic loosening

**Figure 1.**
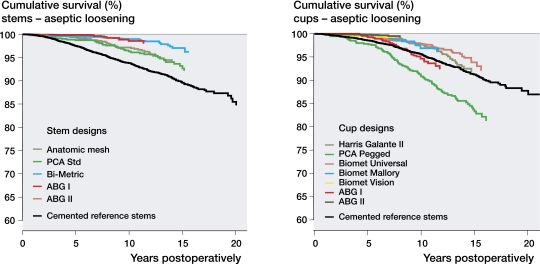
Cox-adjusted survival curves for 19,859 stems and 19,859 cups in patients aged 55 years or older with stem designs (panel A) or cup designs (panel B) as the strata factors. The endpoint was defined as stem (A) or cup (B) revision due to aseptic loosening. Adjustment was made for age and sex. For an explanation of abbreviations, see Table 4.

When all patients aged 55 years or more were analyzed as a single group, the Bi-Metric stem had a higher survival rate at 15 years than the reference group. The Cox regression analysis revealed that all cementless stems studied had a statistically significantly reduced risk of revision during the first 7 years after the primary operation when compared to the reference group (Table 4). Beyond 7 years of follow-up, the Bi-Metric and the ABG I stems still showed significantly lower revision risks than the cemented reference group (Table 4, Figure 1A).

**Table 4.** Survival of cementless stems and the cemented reference group. Endpoint was defined as revision due to aseptic loosening of the stem. 7-, 10-, and 15-year survival rates were obtained from the Kaplan-Meier analysis. For prosthesis types, see Table 1

A	B	C	D	E	F	G	H	I	J	K	L
All (≥ 55 years)											
	Anatomic Mesh	604	11.1	532	98 (97–99)	444	96 (94–98)	91	92 (89–95)	0.53 (0.37–0.76)	0.001
	FU ≤ 7 years									0.38 (0.21–0.71)	0.002
	FU > 7 years									0.64 (0.40–1.00)	0.05
	PCA Std	508	11.6	430	98 (96–99)	351	95 (92–97)	146	89 (86–93)	0.62 (0.44–0.88)	0.007
	FU ≤ 7 years									0.49 (0.27–0.89)	0.02
	FU > 7 years									0.68 (0.44–1.05)	0.08
	Bi-Metric	5,379	6.8	2,698	99 (99–99)	1,463	99 (98–99)	154	96 (94–98)	0.20 (0.15–0.27)	< 0.001
	FU ≤ 7 years									0.18 (0.12–0.26)	< 0.001
	FU > 7 years									0.24 (0.16–0.37)	< 0.001
	ABG I	2,330	6.7	1,152	100 (99–100)	342	98 (97–99)	0	–	0.15 (0.09–0.25)	< 0.001
	FU ≤ 7 years									0.08 (0.04–0.18)	< 0.001
	FU > 7 years									0.40 (0.20–0.80)	0.009
	ABG II	1,489	2.5	0	–	0	–	0	–	0.31(0.13–0.76)	0.01
	FU ≤ 7 years									0.31 (0.13–0.76)	0.01
	FU > 7 years									–	–
	Cemented	9,549	8.8	6,231	97 (96–97)	4,442	95 (94–95)	1,115	91 (90–92)	1.0 (reference)	–
	FU ≤ 7 years									1.0 (reference)	–
	FU > 7 years									1.0 (reference)	–
	Total	19,859									
A Age group											
B Brand of stem											
C Number of operations											
D Mean follow-up (in years)											
E At risk (7-year)											
F % 7-year survival (95% CI)											
G At risk (10-year)											
H % 10-year survival (95% CI)											
I At risk (15-year)											
J % 15-year survival (95% CI)											
K Adjusted RR **^a^** for revision (95% CI)											
L p-value											
**^a^** RR: risk ratio from the Cox regression analysis (other stem brands compared to the cemented reference stems, with adjustment made for age and sex)											

For the age groups 55–64 years and 65–74 years, the Bi-Metric stem had a higher 15-year survival rate than the reference group (95% (CI: 92–97) vs. 84% (CI: 80–87) and 98% (CI: 97–99) vs. 90% (CI: 89–91), respectively).

### Survival of cups – aseptic loosening

When all patients aged 55 years or more were analyzed as a single group, the survival of the PCA Pegged cup at 15 years was lower than that of the reference group. Apart from this exception, there were no differences in survival rates between cementless cups and that of the reference group at 15-years. The Cox regression analysis revealed that the PCA Pegged cup had a significantly increased risk of revision both during the first 7 years postoperatively and beyond 7 years of follow-up. Furthermore, during the first 7 years the Press-Fit Universal, the Mallory, the Vision, and the ABG II cups had significantly reduced risks of revision compared to the reference group (Table 5). Beyond 7 years of follow-up, however, the lower revision risk remained only for the Press-Fit Universal cup (Table 5, Figure 1B). The number of Vision and ABG II cups for analysis beyond 7 years was low (Table 5).

**Table 5.** Survival of cementless cups and the cemented reference group. Endpoint was defined as revision due to aseptic loosening of the cup. 7-, 10-, and 15-year survival rates were obtained from the Kaplan-Meier analysis. For abbreviations, see Table 4

A	B	C	D	E	F	G	H	I	J	K	L
All (≥ 55 years)											
	HG-II	604	11.1	531	99 (98–100)	445	97 (95–98)	92	88 (84–92)	0.75 (0.53–1.05)	0.09
	FU ≤ 7 years									0.50 (0.24–1.03)	0.0
	FU > 7 years									0.82 (0.56–1.22)	0.3
	PCA Pegged	508	11.6	432	94 (92–96)	353	86 (83–89)	148	75 (70–80)	1.91 (1.49–2.44)	< 0.001
	FU ≤ 7 years									2.21 (1.44–3.38)	< 0.001
	FU > 7 years									1.70 (1.26–2.30)	< 0.001
	PFU	2,687	8.8	2,035	98 (97–99)	1,192	97 (96–98)	145	91 (87–94)	0.54 (0.42–0.70)	< 0.001
	FU ≤ 7 years									0.68 (0.47–0.99)	0.04
	FU > 7 years									0.45 (0.32–0.64)	< 0.001
	Mallory	637	8.8	517	99 (98–100)	275	96 (94–98)	10	–	0.52 (0.31–0.86)	0.01
	FU ≤ 7 years									0.35 (0.14–0.85)	0.02
	FU > 7 years									0.68 (0.37–1.26)	0.2
	Vision	2,055	3.4	152	99 (98–100)	0	–	0	–	0.49 (0.27–0.89)	0.019
	FU ≤ 7 years									0.51 (0.27–0.97)	0.04
	FU > 7 years									3.39 (0.47–24.8)	0.2
	ABG I	565	9.1	454	98 (96–99)	330	93 (90–95)	0	–	1.17 (0.83–1.66)	0.4
	FU ≤ 7 years									0.81 (0.43–1.52)	0.5
	FU > 7 years									1.46 (0.96–2.24)	0.08
	ABG II	3,254	4.3	700	99 (99–100)	14	–	0	–	0.20 (0.11–0.38)	< 0.001
	FU ≤ 7 years									0.22 (0.11–0.43)	< 0.001
	FU > 7 years									0.19 (0.03–1.37)	0.1
	Cemented	9,549	8.8	6,221	98 (98–98)	4,441	96 (96–97)	1,113	92 (91–93)	1.0 (reference)	–
	FU ≤ 7 years									1.0 (reference)	–
	FU > 7 years									1.0 (reference)	–
	Total	19,859									

For patients aged 55–64 years, the HG-II cup (87% (CI: 82–91)) and the PFU cup (88% (CI: 84–93)) had similar survival rates at 15 years as the reference cups (85% (CI 81-88)). For patients aged 65–74 years, the PFU cup had higher survival rate at 15 years than the reference group (96% (CI: 94–98) vs. 92% (CI: 91–93)].

### Survival of total hip replacements (cup + stem combinations) – aseptic loosening

**Figure 2.**
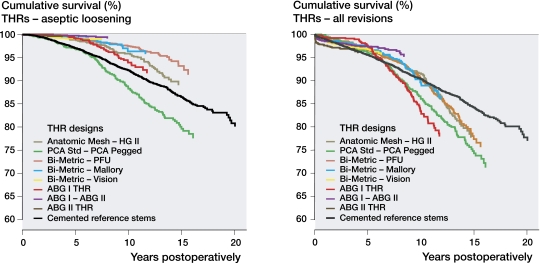
Cox-adjusted survival curves for 19,859 total hip replacements in patients aged 55 years or older with total hip replacement design as the strata factor. The endpoint was defined as revision of the stem and/or the cup due to aseptic loosening (panel A) or as revision for any reason (B). Adjustment was made for age and sex. For an explanation of abbreviations, see Table 4.

When all patients aged 55 years or more were analyzed as a single group, the 15-year survival of the PCA Standard/PCA Pegged was lower than that of the reference group. The Cox regression analysis revealed that the PCA Standard/PCA Pegged had a significantly increased risk of revision beyond 7 years of follow-up. In contrast, all other cementless cup designs showed lower risk of revision than the cemented reference group during the first 7 years, and the Bi-Metric/Press-Fit Universal, the Bi-Metric/Mallory, and the ABG I/ABGII even beyond 7 years (Figure 2A, Table 6). Beyond 7 years, the number of Bi-Metric/Vision THRs was low (Table 6).

**Table 6.** Survival of cementless total hip replacements and the cemented reference group. Endpoint was defined as revision due to aseptic loosening of the cup and/or the stem. 7-, 10-, and 15-year survival rates were obtained from the Kaplan-Meier analysis. For abbreviations, see Table 4

A	B	C	D	E	F	G	H	I	J	K	L
All ≥ 55 years)											
	Anatomic Mesh/HG-II	604	11.1	532	97 (96–98)	445	94 (92–96)	92	85 (80–89)	0.68 (0.51–0.90)	0.006
	FU ≤ 7 years									0.52 (0.32–0.86)	0.01
	FU > 7 years									0.75 (0.53–1.06)	0.1
	PCA Std/PCA Pegged	508	11.6	433	92 (90–95)	354	83 (80–87)	148	71 (66–76)	1.48 (1.19–1.83)	< 0.001
	FU ≤ 7 years									1.37 (0.96–1.96)	0.09
	FU > 7 years									1.46 (1.11–1.92)	0.006
	Bi-Metric/PFU	2,687	8.8	2,035	98 (97–98)	1,192	96 (96–97)	145	90 (87–93)	0.37 (0.29–0.46)	< 0.001
	FU ≤ 7 years									0.39 (0.29–0.54)	< 0.001
	FU > 7 years									0.34 (0.25–0.47)	< 0.001
	Bi-Metric/Mallory	637	8.8	517	99 (98–100)	275	96 (94–98)	10	–	0.36 (0.23–0.58)	< 0.001
	FU ≤ 7 years									0.27 (0.13–0.55)	< 0.001
	FU > 7 years									0.48 (0.26–0.88)	0.02
	Bi-Metric/Vision	2,055	3.4	152	99 (98–99)	0	–	0	–	0.37 (0.23–0.60)	< 0.001
	FU ≤ 7 years									0.37 (0.23–0.62)	< 0.001
	FU > 7 years									2.53 (0.35–18.4)	0.4
	ABG I/ABG I	565	9.1	455	97 (96–99)	330	92 (90–95)	0	–	0.75 (0.54–1.04)	0.09
	FU ≤ 7 years									0.51 (0.29–0.87)	0.01
	FU > 7 years									1.03 (0.68–1.55)	0.9
	ABG I/ABG II	1,765	5.9	700	99 (99–100)	14	–	0	–	0.12 (0.06–0.22)	< 0.001
	FU ≤ 7 years									0.12 (0.06–0.24)	< 0.001
	FU > 7 years									0.13 (0.02–0.93)	0.04
	ABG II/ABG II	1,489	2.5	0	–	0	–	0	–	0.33 (0.15–0.74)	0.007
	FU ≤ 7 years									0.34 (0.15–0.76)	0.009
	FU > 7 years									–	–
	Cemented	9,549	8.8	6,234	96 (96–96)	4,447	93 (93–94)	1,116	88 (87–89)	1.0 (reference)	–
	FU ≤ 7 years									1.0 (reference)	–
	FU > 7 years									1.0 (reference)	–
	Total	19,859									

For patients aged 55–64 years, the 15-year survival rate of the Bi-Metric/Press-Fit Universal was higher than that for the reference group (88% (CI: 84–92) vs. 78% (CI: 74–82)). For patients aged 65–74 years also, the survival rate at 15 years of the Bi-Metric/Press-Fit Universal (95% (CI: 93–98)) was higher than that for the reference group (87% (CI: 86–89%)).

### Survival of total hip replacements (cup + stem combinations) – all revisions

When all patients aged 55 years or more were analyzed as a single group, the survival rate at 15 years of the cementless designs was lower than that for the reference group. The Cox regression analysis revealed that during the first 7 years postoperatively, the ABG I/ABGII had a significantly reduced risk of revision compared to the cemented reference group (Table 7). Furthermore, the ABG II/ABG II combination was the only design to show an increased risk of revision during the first 7 years after the primary operation (Table 7). Beyond 7 years of follow-up, however, several cementless designs (the Anatomic Mesh/HG-II, the PCA Standard/PCA Pegged, and the ABG I/ABG I) showed higher risk of revision than the cemented reference group (Table 7, Figure 2B), and none of the cementless designs had a lower risk of revision than the reference group beyond 7 years. Beyond 7 years, the number of ABG I/ABG II THRs was low (Table 7).During the first 7 years postoperatively in the patients aged 55–64 years, the risk ratio for revision of cementless THRs for any reason was not significantly different from that of the cemented reference group (Table 7). Beyond 7 years of follow-up, however, the revision risk of the Anatomic Mesh/HG-II (RR = 1.6, CI: 1.2–2.2), the PCA Std/PCA Pegged (RR = 1.5, CI: 1.1–2.1), the Bi-Metric/PFU (RR = 1.5, CI: 1.1–1.9), the Bi-Metric/Mallory (RR = 2.0, CI: 1.3–3.1), and the ABG I/ABG I (RR = 3.2, CI: 2.3–4.6) was higher than that of the reference group.

**Table 7.** Survival of cementless total hip replacements and the cemented reference group. Endpoint was defined as revision of the cup and/or the stem for any reason. 7-, 10-, and 15-year survival rates were obtained from the Kaplan-Meier analysis. For an explanation of abbreviations, see Table 4

A	B	C	D	E	F	G	H	I	J	K	L
55–64											
	Anatomic Mesh/HG-II	385	11.2	343	94 (92–97)	296	89 (86–92)	57	70 (64–76)	1.18 (0.91–1.54)	0.2
	PCA Std/PCA Pegged	347	12.2	303	91 (88–94)	262	82 (78–86)	119	66 (61–72)	1.32 (1.03–1.69)	0.03
	Bi-Metric/PFU	1,863	9.1	1,488	93 (92–94)	872	86 (85–88)	102	66 (61–72)	1.18 (0.97–1.44)	0.09
	Bi-Metric/Mallory	266	9.0	224	95 (92–97)	119	83 (77–88)	8	72 (61–82)	1.27 (0.90–1.80)	0.2
	Bi-Metric/Vision	1,080	3.8	96	95 (93–97)	0	–	0	–	1.05 (0.72–1.54)	0.8
	ABG I/ABG I	280	9.4	239	90 (87–94)	174	74 (69–80)	0	–	2.13 (1.62–2.81)	< 0.001
	ABG I/ABG II	746	6.0	309	95 (93–97)	6	–	0	–	0.90 (0.62–1.31)	0.6
	ABG II/ABG II	610	2.4	0	–	0	–	0	–	1.54 (0.93–2.53)	0.0
	Cemented	1,204	9.5	837	92 (91–94)	616	89 (87–91)	210	76 (72–79)	1.0 (reference)	–
	Subtotal	6,781									
65–74											
	Anatomic Mesh/HG-II	186	11.3	165	94 (90–97)	133	90 (86–95)	34	77 (67–87)	1.05 (0.70–1.56)	0.8
	PCA Std/PCA Pegged	133	11.0	112	91 (86–96)	85	81 (74–88)	30	70 (61–80)	1.78 (1.23–2.58)	0.002
	Bi-Metric/PFU	740	8.5	525	94 (92–96)	320	90 (87–93)	45	85 (81–89)	0.89 (0.68–1.16)	0.4
	Bi-Metric/Mallory	274	8.8	223	95 (92–98)	119	91 (87–95)	2	–	0.88 (0.57–1.34)	0.5
	Bi-Metric/Vision	850	3.1	51	94 (90–97)	0	–	0	–	1.10 (0.74–1.63)	0.6
	ABG I/ABG I	238	9.0	187	91 (87–95)	142	86 (81–91)	0	–	1.34 (0.93–1.92)	0.1
	ABG I/ABG II	789	5.9	311	98 (96–99)	8	–	0	–	0.48 (0.31–0.75)	0.001
	ABG II/ABG II	647	2.4	0	–	0	–	0	–	1.62 (1.05–2.51)	0.03
	Cemented	4,964	9.3	3,446	94 (93–95)	2,635	90 (89–91)	722	85 (83–87)	1.0 (reference)	–
	Subtotal 8,821										
>75											
	Anatomic Mesh/HG-II	33	8.9	25	90 (80–100)	17	–	2	–	1.69 (0.54–5.32)	0.4
	PCA Std/PCA Pegged	28	7.6	18	–	8	–	1	–	2.44 (0.78–7.66)	0.1
	Bi-Metric/PFU	84	5.6	31	93 (86–99)	14	–	1	–	1.60 (0.65–3.90)	0.3
	Bi-Metric/Mallory	97	8.1	73	98 (95–100)	39	1	–		0.45 (0.11–1.80)	0.3
	Bi-Metric/Vision	125	2.8	6	–	0	–	0	–	2.09 (0.92–4.77)	0.08
	ABG I/ABG I	47	8.0	33	100 (100–100)	21	100 (100–100)	0	–	–	–
	ABG I/ABG II	230	5.6	82	97 (94–99)	1	–	0	–	0.70 (0.31–1.60)	0.4
	ABG II/ABG II	232	2.6	0	–	0	–	0	–	1.69 (0.85–3.35)	0.1
	Cemented	3,381	7.6	1,954	96 (95–97)	1,198	95 (94–96)	186	94 (92–95)	1.0 (reference)	–
	Subtotal	4,257									
All (≥ 55 years)											
	Anatomic Mesh/HG-II	604	11.1	532	94 (92–96)	446	89 (87–92)	93	72 (67–78)	1.19 (0.97–1.47)	0.1
	FU ≤ 7 years									0.91 (0.64–1.29)	0.6
	FU > 7 years									1.32 (1.01–1.72)	0.04
	PCA Std/PCA Pegged	508	11.6	433	91 (89–94)	354 82 (79–86)	149	67 (62–72)	1.51 (1.24–1.83)	< 0.001	
	FU ≤ 7 years									1.26 (0.91–1.75)	0.2
	FU > 7 years									1.52 (1.18–1.95)	0.001
	Bi-Metric/PFU	2,687	8.8	2,044	93 (92–94)	1,205	87 (86–89)	147	71 (67–75)	1.10 (0.95–1.27)	0.2
	FU ≤ 7 years									0.98 (0.80–1.20)	0.8
	FU > 7 years									1.18 (0.96–1.46)	0.1
	Bi-Metric/Mallory	637	8.8	519	95 (93–97)	277	89 (85–92)	10	–	1.05 (0.81–1.36)	0.7
	FU ≤ 7 years									0.76 (0.52–1.12)	0.2
	FU > 7 years									1.43 (1.00–2.05)	0.05
	Bi-Metric/Vision	2,055	3.4	153	95 (93–96)	0	–	0	–	0.99 (0.76–1.29)	0.9
	FU ≤ 7 years									0.98 (0.75–1.29)	0.9
	FU > 7 years									2.75 (0.38–19.9)	0.3
	ABG I/ABG I	565	9.1	458	91 (89–94)	336	81 (77–84)	0	–	1.74 (1.41–2.15)	< 0.001
	FU ≤ 7 years									1.27 (0.92–1.74)	0.1
	FU > 7 years									2.30 (1.73–3.06)	< 0.001
	ABG I/ABG II	1,765	5.9	701	96 (95–97)	14	–	0	–	0.66 (0.51–0.86)	0.002
	FU ≤ 7 years									0.63 (0.48–0.84)	0.001
	FU > 7 years									0.69 (0.28–1.69)	0.4
	ABG II/ABG II	1,489	2.5	0	–	0	–	0	–	1.52 (1.13–2.05)	0.006
	FU ≤ 7 years									1.46 (1.08–1.97)	0.01
	FU > 7 years									–	–
	Cemented	9,549	8.8	6,237	94 (94–95)	4,449	91 (91–92)	1,117	86 (84–87)	1.0 (reference)	–
	FU ≤ 7 years									1.0 (reference)	–
	FU > 7 years									1.0 (reference)	–
	Total	19,859									

During the first 7 years in the patients aged 65–74 years, the risk ratio for revision due of cementless THRs for any reason was not significantly different from that of the cemented reference group, except that the ABG I/ABG II had a reduced risk of revision compared to that of the reference group (RR = 0.45, CI: 0.28–0.72). Beyond 7 years of follow-up, the risk ratio for revision due of cementless THRs for any reason was not significantly different from that of the cemented reference group, except that the PCA Std/PCA Pegged (RR = 2.0, CI: 1.3–3.3) had an increased risk of revision compared to the reference group.

## Discussion

We found that the survival rate for aseptic loosening of the best performing cementless stems in patients aged 55–74 years was higher than that of the cemented reference stems. Biological fixation in itself seems to be a reliable method in THA of elderly patients. However, the survival rate of the cemented reference implants for any reason was higher than that of cementless implants. Polyethylene wear and osteolysis remain a serious problem with all cementless cup designs with unplugged screw-holes and poor liners. A longer follow-up is required in order to determine whether cups with plugged screw-holes and modern liner options provide any solution to the wear-problem.

Registry-based studies have certain limitations. The coverage of the Finnish Arthroplasty Register before the period 1994–1995 was only 90% (Puolakka et al. 2001a). The missing 10% of implant data may have caused bias in our study. It is also possible that only a few centers performed most of the implantations of certain designs. However, a single center with poor results would be unlikely to have a major effect on the results in a study with such a high number of implants. Moreover, it is the purpose of registry studies to evaluate population-based results, including hospitals of variable standards. Another possible limitation of registry-based studies is their single definition of failure, i.e. a revision operation. There may be patients with osteolysis or loosened implants who are too ill to undergo revision surgery or who simply prefer not to do so. Furthermore, the adjustments in the Cox model in our study were performed only for 2 confounders: age and sex. Many other potential confounders, such as antibiotic prophylaxis or hospital operative volume, may be associated with the relationship between implant brand and revision rate.

The implant designs varied over the long study period (Table 1). Some of the 3 cemented designs we used as the reference group were implanted over the whole study period, starting in 1980. Any recent developments in cementing techniques that were adopted may have resulted in higher long-term survival rates for those prostheses that were implanted later in the study period (Herberts and Malchau 2000, Malchau et al. 2002). However, the cemented implants we chose were the best performing designs in the Finnish Register regardless of the time period they were implanted (Mäkelä et al. 2008b).

The proportional hazards assumption of the Cox model (meaning that the relative difference between revision rates should be constant over time since the primary operation) was not reached in some analyses. Thus, adjusted risk ratios were also established within time intervals (0–7 years and > 7 years after the primary operation). Follow-up beyond 7 years revealed that the results of cementless cups, and therefore of cementless THRs, detoriate with time.

The revision rates that we found for cementless implants were similar to previous findings (Malchau et al. 1997, Thanner et al. 1999, Xenos et al. 1999, Archibeck et al. 2001, Puolakka et al. 2001b, Giannikas et al. 2002, Bojescul et al. 2003, Jacobsen et al. 2003, Duffy et al. 2004, Herrera et al. 2004, Marshall et al. 2004, Moskal et al. 2004, Oosterbos et al. 2004, Kim 2005, Eskelinen et al. 2006, Castoldi et al. 2007, Firestone et al. 2007, Surdam et al. 2007).

We found a higher long-term survival rate for the Bi-Metric stem than for the reference stems in patients aged 55–74 years. When revisions for aseptic loosening were analyzed, the Press-Fit Universal cup was found to have a long-term survival rate similar to those of the reference cups in patients aged 55–74 years. In Finland, Biomet cups were used with Hexloc liners until 1995, and with Ringloc liners after that. In an earlier study based on data from the Finnish Register, survivorship of the Press-Fit Universal cups with Hexloc liners was poor (Puolakka et al. 1999). Reasons for increased wear of Hexloc liners were thin polyethylene, poor quality of the polyethylene, cylindrical design, and a poor locking mechanism (Puolakka et al. 1999, 2001b). Furthermore, the screw-holes of Press-Fit Universal cups were unplugged. In the present study, the survival rate of the Bi-Metric/Press-Fit Universal at 15 years was lower than that of the cemented reference group when all revisions were taken into account. However, the adjusted risk of revision of the Bi-Metric/Press-Fit Universal for any reason was similar to that of the reference group. This finding is probably influenced by the positive effect of Ringloc liners (starting in 1995) on the results with the Bi-Metric/Press-Fit Universal. Unfortunately, it is not possible to analyze the survival rate of the Press-Fit Universal cups with Hexloc liners and with Ringloc liners separately in the Finnish Register data. Revision risk for any reason with the Bi-Metric/Vision was similar to that for the cemented reference group (Table 7, Figure 2B). However, survival rates at 10-years for the Vision cup with Ringloc-liners and plugged screw-holes are not yet available.

The survival rate for aseptic loosening of the Anatomic Mesh/Harris-Galante II at 15 years was not significantly different from that of the cemented reference group. Nonetheless, the survival rate of the Anatomic Mesh/HG-II for any reason at 15 years was poor. Again, this finding can be attributed to wear-related factors. The Anatomic Mesh/Harris-Galante II is no longer being implanted in patients in Finland.

The 15-year survival rate for the PCA Standard stem in our study was lower than those for the best-performing stems. The PCA Standard/PCA Pegged prosthesis is no longer being implanted in patients in Finland.

The 10-year survival rate of the ABG I/ABG I for any reason was lower than that for the reference group. However, the survival rate of the ABG I stem at 10 years for aseptic loosening was higher than that for the reference group. For this reason, and because of poor liners in the ABG I cup design, in Finland the ABG I stem has been widely used along with the ABG II cup with plugged screw-holes and thicker Duration liners consisting of stabilized polyethylene (Stryker, Mahwah, NJ). In our study, the risk of revision of the ABG I/ABG II for any reason in patients aged 65–74 was lower than that for the reference group when all revisions were taken into account (Table 7, Figure 2B). However, the survival rates for the ABG I/ABG II at 10 years are not yet available. Survivorship of modular cementless cups may dramatically worsen after 7–10 years of follow-up due to excessive wear and osteolysis, as indicated by the beyond-7-years survival analysis in our study. Thus, it is too early to draw any definite conclusions about the long-term success of this hip implant.

The ABG II stem differs from the ABG I stem regarding its titanium alloy composition, its stem geometry, its macrotexture, its conus size, and the option with Zirkonia heads (ABG II Cement-Free Hip System). The risk of revision of the ABG II/ABG II for any reason was higher than that for the reference group. The mean follow-up time for the ABG II/ABG II was short: only 2.5 years (Table 1). The proportion of periprothetic fractures for all revisions of the ABG II/ABG II was high: 37% (Table 3). This finding is in accordance with clinical experience in Finland. The ABG II stem appears to be vulnerable to perioperative periprothetic femoral fractures, due to its anatomical and conical shape. There were only 3 aseptic loosenings of the ABG II stem during the study period (Table 3). The problem with an early aseptic loosening of a cementless stem is that there may not have been any osseointegration at all from the beginning, due to undersizing or some other technical failure. Thus, strictly speaking any associated loosening could not have happened either. A longer follow-up time is needed to determine whether either the ABG I/ABG II or the ABG II/ABG II provides a long-term solution to the wear problem. Only a few Zirkonia head or liner fractures have been reported in Finland (Table 3).

For patients aged 75 years and older, the survival rates were similar between cementless implants and the cemented reference group, except that the PCA Pegged cup had an increased risk of revision compared to the cemented reference group. This is in accordance with the results of a previous report from the Finnish Arthroplasty Register (Mäkelä et al. 2008a). However, there was little information for this subgroup.

In conclusion, cementless, proximal porous-coated stems are a good option for elderly patients. Polyethylene wear and osteolysis remain a problem for cementless designs with unplugged screw-holes and low-quality liners.
